# The Role of Parenting Styles in Schoolchildren’s Homework Motivation, Homework Behavior, and Academic Achievement

**DOI:** 10.11621/pir.2025.0404

**Published:** 2025-12-15

**Authors:** Tamara O. Gordeeva, Darina M. Nechaeva, Oleg A. Sychev

**Affiliations:** a Lomonosov Moscow State University, Russia; b Federal Scientific Center for Psychological and Interdisciplinary Research, Moscow, Russia; c Moscow Institute of Psychoanalysis, Russia

**Keywords:** homework motivation, parental autonomy support and control, self-determination theory, persistence, cheating, academic achievement, high school students

## Abstract

**Background.:**

Previous research highlights the importance of parental support, and in particular parental autonomy support, in homework motivation. However, the joint impact of parental autonomy support and parental control on homework motivation, cheating, persistence, and GPA has not been studied, despite the importance of homework motivation and homework behavior for students’ academic achievement.

**Objective.:**

To investigate how students’ parental control and parental autonomy support are related to homework motivation and homework behaviors (e.g., cheating and persistence), and academic achievement in Russian high school students.

**Design.:**

The participants were high school students, *N* = 526 (257 girls and 269 boys, M_age_ = 16.48, *SD* = .61). The measures used included the Perceived Parental Autonomy Support Scale (Mageau et al., 2015), the Motivation for Homework Scale (Gordeeva & Sychev, 2025b), the Homework Behavior Scale (Gordeeva & Sychev, 2025a), and students’ GPA.

**Results.:**

In contrast to control, parental autonomy support showed a clear positive effect on student academic achievement. Structural equation modeling showed that perceived parental autonomy support in childhood is positively associated with students’ GPA, autonomous homework motivation, and productive homework behavior. On the other hand, controlling parenting style demonstrated ambivalent patterns of results, being a predictor of low persistence, external and introjected homework motivation, with the latter related to higher GPA.

**Conclusion.:**

This study extends previous self-determination theory (SDT) research on parenting styles by examining their role in homework behavior and academic performance, particularly emphasizing the dual importance of promoting parental autonomy support and lowering parental control for homework motivation.

## Introduction

According to surveys analyzed by Patall et al. (2008), teachers and parents believe that homework contributes to children’s performance at school. At the same time, surveys of Russian schoolchildren show that the key problem that worries schoolchildren is an overload of homework. The growing volume of homework is one of the most pressing problems of today’s schoolchildren, the “primary stressor” in their lives, associated with a decrease in academic motivation, an increase in negative attitudes towards school, and demotivation (Arshinskaya, 2014). According to international studies, homework is associated with stress and negative health consequences in modern schoolchildren (Katz et al., 2011; Zhao et al., 2024), especially in schools with high achievers (Galloway et al., 2013). Russian parents report that it is difficult for them to motivate high school students to do homework and indicate that the main diiculty in preparing homework is the lack of the child’s motivation, which they associate with the content of homework that is boring, the same for the whole class, often not checked, and when students copy answers from each other (Uskova, 2017). Accordingly, the topic of motivation for homework, its predictors and consequences is highly topical.

Each country’s educational system has its own peculiarities regarding homework and its consequences. Data from PISA international studies on representative samples of 15-year-old schoolchildren show that the time spent by Russian high school students on homework is one of the highest in the world: Russia ranks second in this indicator (China - 13.8 hours per week, Russia - 9.7, Singapore - 9.4) (OECD, 2012). At the same time, it is significant that the association between homework time and academic success in Russia is very weak (OECD, 2012), unlike, for example, China, Japan, and Singapore. Th at is, time is not an indicator of success. This supports the idea of studying other homework indicators as predictors of learning effectiveness, such as motivation for homework, homework behavior, and the role of parents. Research shows the importance of the role of parents in supporting homework motivation (Katz et al., 2011).

To date, motivation for activity, motivation types, their antecedents and consequences have been theoretically developed within the framework of self-determination theory, with many empirical studies supporting the theory, including in the educational context (Ryan & Deci, 2020). The results of meta-analytic studies of academic motivation indicate that intrinsic and identiied motivation are associated with persistence and academic performance, and motivation is negatively associated with low academic achievement (objective data), absenteeism, anxiety, depression, negative emotions and low self-esteem, while introjected motivation occupies an intermediate position and demonstrates weak associations with persistence (Howard et al., 2021).

Previous studies on autonomous motivation in different subjects (Guay & Bureau, 2018) suggest that information about the types of motivation for doing homework will be an important addition to information about the proile of academic motivation. Homework, although part of the general educational process, has unique features, as it is performed in a diferent environment (at home), requires more self-regulation, and could be accompanied by parental involvement. Accordingly, the reasons that motivate children and adolescents to work in the classroom and at home may differ, as well as the antecedents and consequences of homework motivation for academic achievement and successful learning.

Even though there are dozens of studies devoted to diferent types of academic motivation in the classroom and their consequences (Howard et al., 2021), the motivation for doing homework has only just begun to be studied. The first studies on homework motivation showed that autonomous motivation decreases from elementary to high school (4th and 8th grades), and this decrease is associated with the perception of teachers as less supportive of basic psychological needs regarding homework (Katz et al., 2009). Studies have also shown a relationship between autonomous homework motivation and cognitive engagement in high-quality homework (Núñez et al., 2019), as well as less procrastination regarding homework completion and higher self-efficacy (Katz et al., 2014).

A very small number of studies addressed controlled homework motivation and its consequences. This type of motivation has only been explored in a study by Katz etal. (2011). However, a limitation of this study is the joint consideration of controlled motivation, which does not allow an understanding of the speciic contributions of its components — introjected and external motivation to homework behavior.

These results indicate that the type of homework motivation is important. However, the relationship between homework motivation and persistence, cheating, and academic achievement, and the role of parental autonomy support and controlling parenting in these relationships have not been studied yet.

The role of parents in homework motivation and attitudes towards homework has been studied within the constructs of parental involvement (Xu, 2023, 2024) and parental support (Núñez et al., 2015). It is important to note that the results in parental involvement research were mixed. For example, weak positive efects of parental support (Slinakas & Kikas, 2019) and a negative impact of parental support in math performance (Puklek Levpuscek & Zupancic, 2008) were demonstrated. Some studies reported a positive relationship between parental involvement and achievement (Kim, 2022; Pomerantz & Eaton, 2001), whereas others found a negative relationship (Cooper et al., 2000; Patall et al., 2008), and some had mixed results (Dumont et al., 2012, Silinskas & Kikas, 2019). These mixed results are most likely due to the different definitions of parental support as well as parental involvement in homework (Dumont et al., 2012; Grolnick & Pomerantz, 2022; Patall et al., 2008). However, even after clarifying the constructs, some types of involvement really have different consequences, as in the case of parental help with homework (Barger et al., 2019; Moroni et al., 2015).

Recent studies and analyses suggest that the quality of parents’ involvement in their children’s homework may be more important than the quantity of this involvement. Pomerantz et al. (2007) suggested that “how” parents get involved with their children’s homework largely determines the success of this involvement. Four main dimensions that characterize the quality of parents’ involvement in homework are: autonomy support vs. control, process vs. person focus, positive vs. negative afect, and positive vs. negative beliefs about the child’s potential. They concluded that parental involvement may be particularly beneicial for children when it is autonomy-supportive, process focused, characterized by positive afect, and accompanied by positive beliefs. In this study we concentrate primarily on this first dimension — parental autonomy support vs. control — because only this dimension has comprehensive theoretical background that relates parental behavior and practices with students’ motivation and other important outcomes. It is self-determination theory that provides such a theoretical framework. For example, based on SDT, Xu’s study (2025) showed a bidirectional positive association between teacher’s autonomy support for homework and time management. Feng et al. (2019) found that middle school students’ perceived parental autonomy support positively predicted students’ homework autonomous motivation and effort.

Some research suggests that parental control in homework is associated with detrimental efects for academic achievement and attitudes toward school, while parental autonomy support enhances children’s educational outcomes, including academic performance (Dumont et al., 2012, 2014; Karbach et al., 2013; Moroni et al., 2015; Silinskas & Kikas, 2019). A recent study that linked parental control with intrinsic homework motivation has shown that these variables are negatively related to each other (Avci et al., 2025). But no study linked parental control to extrinsic homework motivation. The previous results on parental homework control are mixed. For example, studies have demonstrated the small negative impact of parental homework control on homework expectancy beliefs (Trautwein & Lüdtke, 2009); the high negative impact of parental control on persistence and academic performance (in math) (Sil-inskas & Kikas, 2019); and a positive association of parental homework control with time spent on homework completion, homework time management, and amount of homework completed, but negative associations with academic achievement in math, social sciences, native language and foreign language (Núñez et al., 2015). In the study by Karbach et al. (2013), parents’ autonomy support had no predictive value in academic achievement over general cognitive ability, while high levels of achievement-oriented control and structure were detrimental to academic success.

Research based on self-determination theory (Ryan & Deci, 2000) has differentiated between two types of parental homework involvement and assistance: controlling and autonomy-supportive, which correspond to two main dimensions of parenting style — autonomy support and control (Grolnick & Pomerantz, 2022; Pomerantz et al., 2007). The significance of parental autonomy support to the intrinsic and autonomous academic motivation of schoolchildren is evidenced by many studies (Vasquez et al., 2016), including those with Russian samples (Chirkov & Ryan, 2001). This association is explained by the support for children’s basic psychological needs for autonomy, competence, and relatedness given by parents with an autonomy-supportive parenting style (Bureau et al., 2022). Data from studies on samples of schoolchildren from different countries show that autonomy-supportive parenting is related positively to adolescents’ autonomous motivation and performance, including academic achievement (Chen et al., 2021; Cheung & Pomerantz, 2011; Diaconu-Gherasim & Mäirean, 2016; Gordeeva et al., 2024a; Grolnick & Pomerantz, 2022; Soenens et al., 2007; Vasquez et al., 2016). Controlling parenting, which usually refers in SDT to parenting that is domineering and pressuring (Vansteenkiste et al., 2005), is related to maladaptive adolescent outcomes such as internalizing distress and externalizing problems (Grolnick & Pomerantz, 2009, 2022) and low academic achievement (Diaconu-Gherasim, et al., 2025).

We hypothesized that autonomy-supportive parenting which includes offering choices within certain limits, explaining the reasons behind demands and limits, and accepting and recognizing the child’s feelings (Mageau et al., 2015), would be positively related to autonomous homework motivation, homework behavior, and academic achievement. In particular, it is expected that a significant contribution is made by parental autonomy support to students’ persistence and academic achievement, mediated by autonomous homework motivation. We also hypothesize that parents’ controlling style, operationalized as threatening to punish the child, inducing guilt, and encouraging performance goals would have, in turn, detrimental effects on adolescents’ motivation for homework, homework behavior, and cheating. We controlled for the effects of gender on academic achievement, as previous research consistently shows that female students perform better academically (Rosén et al., 2022), and Russian data conirm these results (Gordeeva et al., 2024a).

## Methods

### Participants and Procedure

We recruited 526 high school students, 10-11^th^ graders, 257 girls and 269 boys, (M_age_ = 16.48; *SD* = .61) from two large Moscow public schools using convenience sampling. The sample size was determined based on a priori power analysis (Lakens, 2022). Based on the studies of the effects of parenting styles discussed in the Introduction, we expected to obtain their effects on homework motivation and behavior of at least moderate magnitude. The results of power analysis showed that for a moderate correlation value of .2, the required sample size was at least 436 participants, so the targeted sample size was about 500. This sample size provided a high level of statistical power (.95) and a conservative significance level of *p* < .01.

The survey was anonymous. All relevant permissions from parents and the Ethical Committee were obtained for the study in accordance with the requirements of the Russian Psychological Society for conducting psychological research.

### Measures

To assess parenting styles, the Perceived Parental Autonomy Support Scale (P-PASS) was used (Gordeeva et al., 2024a; Mageau et al., 2015). The questionnaire begins with the stem phrase “When I was growing up...” and includes two blocks of three scales each. The autonomy support (AS) scale includes three subscales: choice within certain limits (“Choice”); rationale for demands and limits (“Reasons”); and acknowledgement of feelings (“Acceptance”). The controlling parenting (CP) scale includes the following three subscales: threats to punish (“Punishment”); guilt-inducing criticisms (“Guilt”); performance pressures (“Performance”). Each subscale consists of four direct items, for example, “My parents gave me many opportunities to make my own decisions about what I was doing”. Agreement with each statement is rated on a Likert scale from 1 (“Do not agree at all”) to 7 (“Very strongly agree”). For both main scales, the overall scores were calculated as the averages of the scores for the statements included within them. The Cronbach’s α coefficients for this and other measures are shown in *Table 1.*

The homework motivation questionnaire was constructed based on the Academic Motivation Scale (Gordeeva et al., 2014; Vallerand et al., 1992) and motivation to do homework (Katz et al., 2011). Th is questionnaire consists of 13 statements that complete the stem phrase (“I do my homework because...”) and form three scales: autonomous motivation (6 items, *e.g.,* “I like to learn and be able to do more and more”), introjected motivation (3 items, e.g., “I’m ashamed to get bad grades”), and external motivation (4 items, e.g., “I’m not allowed to do anything else until it is done”) (Gordeeva & Sychev, 2025b). Given that autonomous motivation consists of several subtypes demonstrated in previous studies (Vallerand et al., 1992), we included in the relevant subscale three pairs of statements measuring motivation to learn, self-development motivation, and motivation based on the subjective value of learning (identified motivation). A five-point Likert-type scale with response options ranging from 1 (not true) to 5 (true) was used. A three-factor model including covariance within pairs of statements relevant to subtypes of autonomous motivation showed an acceptable fit: χ^2^ = 183.59; df = 59; *p* < .001; CFI = .937; TLI = .916; SRMR = .056; RMSEA = .063 (90% CI = [.053, .074]); PCLOSE = .017; N = 526.

The variables related to students’ behavioral engagement with homework were assessed using the two subscales — the homework persistence scale and the homework cheating scale — from the Homework Behavior Scale (Gordeeva & Sychev, 2025a). In previous studies of homework (e.g., Gordeeva & Sychev, 2025a, 2025b), the construct validity was demonstrated by expected correlations of scales with homework motivation and basic psychological needs satisfaction at school. The factorial validity of the current questionnaire is conirmed by the results of a conir-matory factor analysis of the two-factor model (including covariance between the two reversed items in the homework persistence scale): χ^2^ = 45.31; df= 12; p < .001; CFI = .958; TLI = .927; SRMR = .035; RMSEA = .073; 90% CI for RMSEA: .051-.096; PCLOSE = .044; N = 526.

The homework persistence scale consisted of four items, two of which were reversed (e.g., “I can’t work long and hard on my homework”). Homework cheating was assessed using three items (“I sometimes cheat on my homework, as many students do”, “Sometimes I just cheat on my homework because I don’t have time to do it at home”, “I just cheat on my homework using the Internet and neural networks”). Students rated their agreement with each item using a four-point scale from 1 (never) to 4 (always).

*Students’ academic achievement* was evaluated via the grades on 12 school subjects (all subjects except physical education) for the last term, as reported by the respondent. GPA was calculated as the average of these 12 grades for each student.

A data analysis was carried out using descriptive statistics, a correlation analysis, and Welch’s t-test in the environment for statistical computing R. Structural equation modeling was performed in Mplus 8 with the robust maximum likelihood estimator (MLR). For binary predictor (gender) obtained path coeicients were standardized by the standard deviation for dependent variable (STDY option). A bootstrap analysis in Mplus (5.000 resamples) was used to estimate the significance of mediated effects.

## Results

*Table 1* provides descriptive statistics and correlations for perceived parental styles (autonomy support and controlling parenting), motivation for homework, home-work persistence, cheating, and GPA. As can be seen, GPA correlated positively with parental autonomy support (including its subscales), autonomous motivation and in-trojected motivation, and homework persistence, while correlation with cheating on homework was negative. Autonomous motivation showed positive correlations with autonomy support, homework persistence, and negative correlation with cheating on homework. Introjected motivation was positively related to both autonomous and external motivation, as expected according to its central position in the autonomy continuum. It was also positively related to homework persistence and controlling parenting. As expected, extrinsic motivation was negatively related to perceived autonomy support and positively to controlling parenting.

**Table 1:** Descriptive Statistics and Correlations of All Variables (N = 526).

Measures	1	2	3	4	5	6	7	8	9	10	11	12	13	14
1. GPA	—													
2. Autonomous motivation	.25*	—												
3. Introjected motivation	.24*	.31*	—											
4. External motivation	.00	.00	.39*	—										
5. Homework persistence	.27*	.59*	.22*	-.09	—									
6. Homework cheating	-.21*	-.42*	-.07	.11	-.52*	—								
7. Choice within certain limits (AS)	.15*	.28*	-.10	-.27*	.32*	-.21*	—							
8. Rationale for demands and limits (AS)	.17*	.28*	.02	-.09	.29*	-.21*	.68*	—						
9. Acknowledgement of feelings (AS)	.17*	.30*	-.11	-.25*	.35*	-.26*	.81*	.69*	—					
10. Threats to punish (CP)	-.08	-.22*	.12	.41*	-.33*	.23*	-.56*	-.44*	-.61*	—				
11. Guilt-inducing criticisms (CP)	-.08	-.15*	.15*	.35*	-.30*	.20*	-.59*	-.47*	-.65*	.77*	—			
12. Performance pressures (CP)	.02	.04	.22*	.41*	-.06	.06	-.29*	-.21*	-.35*	.46*	.50*	—		
13. Autonomy support (AS)	.18*	.32*	-.07	-.22*	.35*	-.25*	.91*	.87*	.93*	-.59*	-.63*	-.31*	—	
14. Controlling parenting (CP)	-.06	-.13	.19*	.46*	-.28*	.19*	-.57*	-.44*	-.63*	.88*	.89*	.77*	-.61*	—
Cronbach’s *α*	.88	.85	.80	.69	.71	.78	.81	.76	.85	.89	.87	.76	.92	.90
Means	4.33	3.2	2.69	2.14	2.8	2.51	5.59	5.09	5.06	2.83	2.56	3.63	5.25	3.01
SD	.43	.95	1.19	.93	.69	.84	1.23	1.29	1.43	1.59	1.56	1.54	1.19	1.33

Note. Statistical significance: * P < .001. The numbers of the variables in the columns correspond to their numbers in the rows.

The subscales and main scales of the Perceived Parental Autonomy Support questionnaire showed the expected correlations with each other. Within the two main subscales (autonomy support and controlling parenting), the correlations were positive and significant, and the correlations between the subscales of different blocks were negative. Accordingly, the correlation between the overall scores for the autonomy support and controlling parenting scales was expectedly negative and high in magnitude.

There was a significant effect of gender on GPA (*t*(524) = 5.06, *p* < .001, Cohen’s *d* = .44): girls demonstrated higher GPA (*M* = 4.42, *SD* = .41) than boys (*M* = 4.24, *SD* = .43). Scores on introjected motivation were also significantly higher in girls (*M* = 2.97, *SD* = 1.25) compared to boys (*M* = 2.42, *SD* = 1.07), *t*(504) = 5.46, *p* < .001, Cohen’s d = .48. Cheating on homework was also higher in girls (*M* = 2.62, *SD* = .82) compared to boys (*M* =2.41, *SD* = .85), t(524) = 2.82, *p* < .01, Cohen’s d = .25. Finally, scores on perceived parents’ guilt-inducing criticisms were higher in girls (*M* =2.74, *SD* = 1.66) compared to boys (*M* = 2.39, *SD* = 1.44), *t*(506) = 2.62, *p* < .01, Cohen’sd = .23.

Boys scored slightly higher on homework persistence (*M* = 2.87, *SD* = .68) compared to girls (*M* = 2.73, *SD* = .70), *t*(520) = 2.28, *p* < .05, Cohens *d* = .2. Th ere was also signiicant efect of gender on autonomy support (and all its subscales): *t*(508) = 3.17, *p* < .01, Cohen’s *d* = .28, boys scored higher (*M* =5.41, *SD* = 1.10) than in girls (*M* =5.08, *SD* = 1.26). Scores on performance pressures were slightly higher in boys (*M* = 3.76, *SD* = 1.38) compared to girls (*M* =3.49, *SD* = 1.68), *t*(495) = 2.02, *p* < .05, Cohen’s *d* = .18.

**Figure 1.**
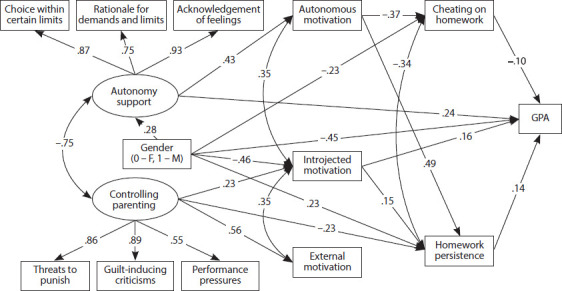
Structural equation model of the relationships between parenting styles, gender, homework motivation, homework persistence, cheating on homework, and academic achievements (residuals, insignificant paths and coefficients are omitted for the sake of parsimony; all coefficients shown in the model are standardized and significant at p < .05)

To examine the efects of parental autonomy support and controlling parenting on homework motivation, homework persistence, cheating on homework, and academic performance, we tested a structural model. In this model the indicators of homework motivation, persistence, cheating, and GPA were considered depending on factors of parental autonomy support and controlling parenting. In addition, cheating on homework, persistence and GPA depended on motivation indicators. We also expected to find the effects of homework cheating and persistence on GPA. The model included gender as a controlled variable with all its possible efects on the other variables and factors. Testing this structural model revealed its acceptable it to the data: χ^2^ = 121.82; *df* = 36; *p* < .001; CFI = .965; TLI = .925; SRMR = .041; RMSEA = .067 (90% CI = [.054, .081]); PCLOSE = .015; *N* = 526. *Figure 1* shows the statistically sig-niicant relationships in this model.

The model presented in *Figure 1* shows that homework autonomous motivation depended on parental support, while controlled motivation (introjected and external) depended on controlling parenting. At the same time, parental autonomy support also showed a direct positive effect on GPA, and controlling parenting had a negative direct effect on persistence. Of the motivational variables, only introjected motivation showed a direct positive effect on GPA. Persistence, which was directly related to autonomous and introjected motivation, showed a positive effect on GPA, while cheating, which was negatively related to autonomous motivation, had a negative effect.

The effect of gender on academic achievements was relatively strong and significant: its value of -.45 means that the average GPA of girls was .45 standard deviations higher than that of boys. As can be seen from the model, gender was associated also with support for autonomy and persistence (higher in boys), as well as introjected motivation and cheating (higher in girls).

An analysis of the indirect effects of autonomy support and controlling parenting on GPA (*Table 2*) demonstrated that there was a statistically signiicant total indirect effect of autonomy support (.046, *p* < .01) mediated by autonomous motivation, homework persistence, and cheating. An effect of autonomy support on cheating through autonomous motivation was also significant (-.16, *p* < .001) as well as a similar effect on persistence (.21, *p* < .001). There was also a positive indirect effect of controlling parenting on persistence through introjected motivation (.035, *p* < .05). However, in the latter case, this weak positive indirect effect was outweighed by a stronger negative direct effect (-.23, *p* < .001). Thus, controlling parenting did not have a significant impact on the GPA because of its contradictory effects on introjected motivation and persistence, which are equally important for academic performance. In contrast to controlling parenting, parental autonomy support demonstrated an unambiguous positive efect on student academic performance.

**Table 2:** Results of the Bootstrap Analysis of Indirect Effects

Effects	Estimate	P-Value
Indirect effects of parental autonomy support on cheating	-.160	<.001
Indirect effects of parental autonomy support on persistence	.211	<.001
Indirect effects of controlling parenting on persistence	.035	.022
Indirect effects of parental autonomy support on GPA		
Sum of indirect effects	.046	.003
Specific indirect 1: through autonomous motivation and cheating	.017	.065
Specific indirect 2: through autonomous motivation and persistence	.029	.025
Indirect effects of controlling parenting on GPA		
Sum of indirect effects	.042	.017
Speciic indirect 1: through introjected motivation and persistence	.005	.096
Speciic indirect 2: through introjected motivation	.037	.026

## Discussion

Our study further supports the research based on self-determination theory on parental autonomy support and its role in student academic motivation and success (Chirkov & Ryan, 2001; Gordeeva et al., 2024b; Soe, et al., 2025; Teuber et al., 2022) and in adaptive homework functioning (Katz et al., 2011; Xu, 2025). In particular, parental autonomy support, which included choice within certain limits, explaining the rationale for demands and limits, and the acknowledgement of feelings, showed an efect on student performance that was mediated by autonomous homework motivation. Structural equation modeling shows that parental autonomy support during childhood is positively associated with high school students’ academic achievement, and this relationship is partially mediated by autonomous homework motivation as well as productive homework behavior such as persistence and low cheating. Being based on an older sample of students from different academic settings, our study continues the line of previous studies (Feng et al., 2019) that found that middle school students perceived parental autonomy support positively predicted homework autonomous motivation and efort. Overall, our results support the hypotheses put forward and are in line with previous research in SDT on parental autonomy support (Bureau et al., 2022; Vasquez et al., 2016) and extends this research on homework motivation and homework behavior.

Our results are consistent with previous SDT research that autonomy-supportive and controlling parenting practices are highly and inversely related to each other (Gordeeva et al., 2024a; Soenens et al., 2007). The study’s novelty and advantages are based on a new comprehensive conceptualization of the controlling parenting style (Mageau et al., 2015), which includes the tendencies to threaten a child with punishment, induce feelings of guilt, and encourage performance goals. Considering previous mixed results in the homework domain (Núñez et al., 2015) results concerning controlling parenting and its relationship with the studied variables deserve special interest. The results demonstrate positive effects of parental control on external motivation and negative efects on persistence, and controlling parenting is also associated with introjected homework motivation, which in turn is positively associated with GPA. These findings on positive effects of introjected regulation are at odds with those previously reported in the literature (Vansteenkiste, et al., 2005) and may be culturally speciic, related to the speciics of the Russian education system and grading. Previous research shows that Russian parents tend to be controlling (Chirkov & Ryan, 2001) and critical to their children’s achievements (Elliott et al., 2005), so adolescents may develop tolerance to this practice, and it could lead to several positive consequences, as was shown in some cultural contexts (Vansteen-kiste et al., 2005).

The effects of controlling parenting style and its components on the adaptive functioning of adolescents from different cultures are being actively discussed (Brad-shaw et al., 2024; Chen et al., 2016; Soenens et al., 2015). This is consistent with the fact that SDT recognizes both the universal aspects and possible specific influences of parenting styles across cultures. On the other hand, it is worth recognizing that controlling parenting has still not been suiciently researched, and it is possible that it does have some positive consequences, especially related to children’s homework motivation, persistence, and achievements. They help to explain why parents tend to use controlling strategies to regulate child’s homework behavior and academic motivation.

At the same time, the type of control may be critically important: as Silinkas and Kikos (2019) show, the psychological control that includes manipulation, shaming, and threats of punishment may have fewer negative efects on homework motivation and academic performance than behavioral parental control, which includes parents’ intervention in homework and imposition of help. This may also explain the mixed efects of parental control in our study, as control variables include both psychological and behavioral types of control.

Thus, the effects of control turned out to be significantly less clear-cut and not as negative as expected in accordance with SDT and similar studies on a sample of Russian university students (Gordeeva et al., 2024a). This is the basis for further research on the role of controlling parenting and its components in the adaptive functioning of children and their academic achievement.

The data also indicate rather impressive gender differences in variables related to homework motivation, GPA, and parenting style, which has been partly noted in previous studies. For example, the speciics of parental involvement in homework with children of different sexes was previously reported (Cooper et al., 2000). We have found that adolescent girls, despite being more successful at school, perceive parents as less autonomy-supportive and demonstrate more cheating and especially stronger introjected regulation for homework. It is possible that this is also a culturally specific finding related to the peculiarities of upbringing in Russian families, when a girl who is unsuccessful in her studies is considered a failure, and an unsuccessful boy may continue to be perceived as capable and talented, with a great future.

## Conclusion

The study results reveal the important effects of perceived parental autonomy support and control on the homework motivation, homework behavior and academic achievement of high school students. Parental autonomy support during childhood is positively associated with students’ academic achievement, and this relationship is mediated by autonomous homework motivation as well as productive homework behavior such as persistence and low cheating. On the other hand, controlling parenting style is a predictor of both external and introjected homework motivation, with latter associated with higher persistence and GPA. The theoretical value of the study lies in the demonstration of specific effects of introjected regulation on homework persistence and GPA, which are distinct from the effects of external regulation.

Impressive gender differences were found in the variables studied. While these findings provide insight into the sources of gender differences in academic performance, they also raise new questions. Russian girls perceive parents as less autonomy-supportive and demonstrate stronger introjected regulation for homework, more cheating behavior, and higher GPA. On the other hand, rather paradoxically, Russian boys demonstrate more perceived parental autonomy support, less introjected motivation, together with less tendency to cheat and greater persistence in homework, despite significantly lower GPA. These findings merit further investigation.

The practical significance of the study lies in the importance of parental behavioral practices during childhood, particularly autonomy support and controlling parenting, on the subsequent homework motivation, homework behavior, and academic achievement of schoolchildren.

## Limitations and Future Research

In this study, there are some limitations that could be improved upon with further research. First, this study is cross-sectional, and this design does not allow us to draw unambiguous causal conclusions about the effects of parent-child relationships, in particular autonomy support and controlling parenting in childhood, on students’ homework motivation, homework behavior, and academic performance. Other interpretations are also possible. For example, high school students may have distorted memories of their childhoods, considering later experiences and how they feel and experience their relationships with their parents today. Therefore, it is important to understand these data within the context of perceived parent-child relationships.

The second limitation is related to only considering indicators of parenting style reported by students, not by their parents. Future research would beneit by examining assessments of parenting style reported by both students and their parents.

The third limitation of the study is the nature of the sample, which included sufficiently prepared and motivated students selected for high school. Therefore, the findings cannot be directly generalized to middle and elementary school students. Both the student population and the level of school expectations (including homework) vary across these age groups. Speciic research is needed to clarify the relationship between homework motivation, parental parenting style, and academic performance in these groups. Also further research is needed to verify and clarify the gender differences found in cheating on homework and homework persistence.

Lastly, the relationships between parental autonomy support and control in childhood and student homework motivation, behavior, and achievement are quite interesting, but they must be examined in future studies, because these variables are very distant from each other. In particular, it is important for future research to examine how parental autonomy support and parental control regarding homework mediate these relationships.
